# Whole-Genome Sequencing and Annotation of Clostridium tyrobutyricum Strain Cirm BIA 2237, Isolated from Silage

**DOI:** 10.1128/MRA.00492-19

**Published:** 2019-07-25

**Authors:** Edouard Munier, Hélène Licandro-Séraut, Christine Achilleos, Rémy Cachon, Eric Beuvier

**Affiliations:** a URTAL, INRA, Poligny, France; b UMR Procédés Alimentaires et Microbiologiques, Université Bourgogne Franche-Comté, AgroSup Dijon, PAM UMR A 02.102, Dijon, France; University of Maryland School of Medicine

## Abstract

Clostridium tyrobutyricum is the main bacterial species leading to the late blowing defect, a major cause of spoilage in semihard and hard cheeses. This study reports the complete genome sequencing, assembly, and annotation of *C. tyrobutyricum* strain Cirm BIA 2237, formerly called CNRZ 608, isolated from silage.

## ANNOUNCEMENT

The strict anaerobic endospore-forming bacterium Clostridium tyrobutyricum is known to be the leading cause of the late blowing defect in semihard and hard cheeses (1, 2), which results in huge losses of products and profits in the cheese industry. Spores of *C. tyrobutyricum* contaminate the milk during milking and survive throughout the pasteurization process. Butyric fermentation due to *C. tyrobutyricum* produces butyric acid, hydrogen, and carbon dioxide, resulting in characteristic defects in cheeses, such as late blowing and altered flavor. This metabolic pathway remains poorly understood for *C. tyrobutyricum*. To date, the genome sequence of the type strain ATCC 25755 has been sequenced and annotated (3), but its role in the late blowing defect has not been studied. Some projects of *C. tyrobutyricum* draft genome sequencing from the late blowing defect in cheese have been initiated (4, 5).

We report the whole-genome sequencing, assembly, and annotation of *C. tyrobutyricum* strain Cirm BIA 2237 (formerly CNRZ 608 [6]), belonging to the French National Institute for Agricultural Research collection. This strain was isolated from silage and is known to cause the late blowing defect in semihard and hard cheeses. It was also used as a reference strain in many studies (7–14).

*C. tyrobutyricum* was grown in reinforced clostridial medium (RCM) (Biokar, France) at 37°C in an anaerobic chamber. The genomic DNA isolation and sequencing were performed by Genoscreen, France. Extraction of the genomic DNA was performed using the Qiagen Gentra Puregene kit, and the DNA was quantified by fluorescence using the Qubit double-stranded DNA broad-range (dsDNA BR) kit. The sequencing libraries were prepared with the Illumina Nextera XT sample prep kit and sequenced using an Illumina HiSeq 2 × 100-bp sequencing platform. A total of 10,595,027 raw reads were obtained. Raw data quality analysis was performed with FastQC v0.11.5 (15), and elimination of sequences containing undetermined or small-sized (<60 bp) nucleotides or a Phred score under 30 was performed with Prinseq v0.20.4 (16). The *de novo* assembly, which consists of 93 scaffolds, was performed using SPAdes v3.10.1 (k-mer sizes of 21, 33, 55, and 77) (17). The assembly was verified by calculating the reads mapped back to contigs (RMBC) index using Bowtie v2.1.0 with stringent mapping parameters (18) and by evaluating the depth of scaffold sequencing using SAMtools v1.5 (19). Scaffolds with a value of less than 200 bp were removed. Annotation was performed using Prokka v1.11 (20) with the hidden Markov model (HMM) (21) and UniProt (22) databases by predicting open reading frames (ORFs) and HMMER v3.1b2 (23) with the eggNOG v4.5 (24) database for a supplementary annotation from ORFs.

The strain Cirm BIA 2237 genome is composed of a chromosome of 3.16 Mb with a GC content of 30.75% and does not contain plasmids. It comprises 3,182 coding sequences.

The genome analysis showed that this strain is composed of genes encoding the required enzymes involved in butyric fermentation from lactate and acetate. The genome annotation will ease studies of the mechanisms causing the defect of late blowing during cheese ripening.

### Data availability.

The complete genome sequence of *C. tyrobutyricum* strain Cirm BIA 2237 has been deposited in DDBJ/EMBL/GenBank (25) under the accession number CP038158, and the raw reads have been deposited in the NCBI Sequence Read Archive under the accession number PRJNA524264.
